# Supplementary material for: Causal associations of thyroid function and dysfunction with overall, breast and thyroid cancer: A two-sample Mendelian randomization study
Source: Int J Cardiol. Author manuscript; Available in PMC 2021 Oct 18. (PMC7611848; doi:10.1016/j.ijcard.2020.03.053)
Supplement: Supplementary Information [file EMS133104-supplement-Supplementary_Information.pdf]

International Journal of Cardiology 313 (2020) 99–104

Contents lists available at ScienceDirect

International Journal of Cardiology

journal homepage: www.elsevier.com/locate/ijcard

Circulating interleukins in relation to coronary artery disease, atrial fibrillation and ischemic stroke and its subtypes: A two-sample Mendelian randomization study

Shuai Yuan [a,b], Ang Lin [c,d], Qi-qiang He [e], Stephen Burgess [f,g], Susanna C. Larsson [a,b,*]

[a] Unit of Cardiovascular and Nutritional Epidemiology, Institute of Environmental Medicine, Karolinska Institutet, Stockholm, Sweden
[b] Department of Surgical Sciences, Uppsala University, Uppsala, Sweden
[c] Department of Medicine Solna, Immunology and Allergy Unit, Karolinska Institutet, Stockholm, Sweden
[d] Center for Molecular Medicine, Karolinska Institutet, Stockholm, Sweden
[e] School of Health Sciences, Wuhan University, Wuhan, China
[f] Department of Public Health and Primary Care, University of Cambridge, Cambridge, UK
[g] MRC Biostatistics Unit, University of Cambridge, Cambridge, UK

A R T I C L E I N F O

Article history:
Received 13 February 2020
Accepted 20 March 2020
Available online 21 March 2020

Keywords:
Atrial fibrillation
Coronary artery disease
Immune
Interleukins
Ischemic stroke
Mendelian randomization analysis

A B S T R A C T

Background: The causal role of interleukins (ILs) for cardiovascular disease has not been fully elucidated. We conducted a Mendelian randomization study to investigate the associations of circulating ILs with coronary artery disease (CAD), atrial fibrillation (AF), and ischemic stroke.

Methods and results: Single-nucleotide polymorphisms associated with IL-1β, IL-1 receptor antagonist (IL-1ra), IL-2 receptor subunit alpha, IL-6, IL-16, IL-17 and IL-18 were identified from genome-wide association studies. Summary-level data of the outcomes were obtained from three large consortia. Genetic predisposition to higher IL-1ra levels were significantly associated with CAD. The odds ratio was 1.36 (95% confidence interval (CI), 1.14–1.63; $P = 5.37 \times 10^{-4}$) per one standard deviation increase in IL-1ra levels. Genetically higher IL-6 levels, predicted by a variant in the *IL6R* gene and corresponding to reduced IL-6 bio-function, were significantly inversely associated with CAD and AF. The odds ratios per one standard deviation increase in IL-6 levels were 0.64 (95%CI, 0.54–0.76; $P = 2.22 \times 10^{-7}$) for CAD and 0.70 (95%CI, 0.62–0.80; $P = 1.34 \times 10^{-7}$) for AF. There was a suggestive positive association of IL-1ra with cardioembolic stroke and suggestive inverse associations of IL-6 with any ischemic stroke, cardioembolic stroke, and small vessel stroke, and of IL-16 with CAD. The other ILs were not associated with any outcome.

Conclusions: These results strengthen the evidence that IL-6 inhibition may offer a therapeutic approach for prevention of CAD, AF, and ischemic stroke. In contrast, IL-1 inhibition through raised IL-1ra levels may confer increased risk of CAD and cardioembolic stroke. The role of IL-16 for CAD warrants further investigation.

© 2020 The Authors. Published by Elsevier B.V. This is an open access article under the CC BY license (http://creativecommons.org/licenses/by/4.0/).

1. Introduction

Cardiovascular disease (CVD) is a leading cause of premature death and exerts a great burden on medical system, society and sustainable human development worldwide. World Health Organization has been estimated that 17.9 million people died from CVD in 2016, representing approximately 31% of all global deaths. For a better prevention and management of CVD, the etiology has been studied by seeking or verifying potential CVD-associated risk factors, such as body mass index [1], lifestyle factors [2,3], and certain nutrients [4,5]. Behind those established associations, inflammation plays a vital intermediary role in the pathogenesis of CVD [6].

Interleukins (ILs) are a group of cytokines with important roles in the regulation of immune and inflammatory responses. Immunosuppressive drugs have been proved by Food and Drug Administrations for treatments of certain inflammatory diseases and cancers, such as Anakinra (anti-IL-1) and Sarilumab (anti-IL-6) for rheumatoid arthritis and Tocilizumab (anti-IL-6) for large-cell lung carcinoma and ovarian cancer [7–9]. Epidemiological studies of the associations of IL-6 and IL-1 receptor antagonist (IL-1ra), an endogenous inhibitor of both IL-1α

Abbreviations: AF, atrial fibrillation; CAD, coronary artery disease; CI, confidence interval; GWAS, genome-wide association study; ILs, interleukins; IL-1ra, interleukin-1 receptor antagonist; IL-2ra, interleukin-2 receptor subunit alpha; IL-6, interleukin-6; IL-16, interleukin-16; IL-17, interleukin-17; IL-18, interleukin-18; MR, Mendelian randomization; OR, odds ratio; SNP, single-nucleotide polymorphism.

* Corresponding author at: Institute of Environmental Medicine, Karolinska Institutet, Stockholm, 17177, Sweden; Department of Surgical Sciences, Uppsala University, Uppsala 75185, Sweden.

E-mail addresses: shuai.yuan@ki.se (S. Yuan), ang.lin@ki.se (A. Lin), sb452@medschl.cam.ac.uk (S. Burgess), susanna.larsson@ki.se (S.C. Larsson).

https://doi.org/10.1016/j.ijcard.2020.03.053
0167-5273/© 2020 The Authors. Published by Elsevier B.V. This is an open access article under the CC BY license (http://creativecommons.org/licenses/by/4.0/).

and IL-1β, with risk of coronary artery disease (CAD) and ischemic stroke have reported conflicting results. High circulating levels of IL-6 have been associated with an increased risk of CAD in observational studies [10,11]. Furthermore, a functional variant in the IL-6 receptor (*IL6R*) gene, which associates with higher levels of IL-6 and soluble IL-6 receptor as well as decreased levels of C-reactive protein and fibrinogen (two liver-derived proteins reflecting inflammation status), has been shown to be related to lower risk of CAD [12,13]. No clear association has been found between IL-6 levels and risk of stroke, but studies have generally been small and data on ischemic stroke subtypes with different etiologies are scarce [14]. Genetic upregulation of IL-1ra is associated with decreased levels of IL-6 and C-reactive protein and has been observed to be associated with higher odds of CAD but not ischemic stroke [15,16]. The role of other ILs for CVD has been scarcely investigated and the results are inconsistent [17–22].

Genome-wide association studies (GWASs) of circulating ILs have discovered one or more genetic loci for circulating IL-1β, IL-1ra, IL-2ra, IL-6, IL-16, IL-17, and IL-18 levels [23–26]. We conducted a two-sample Mendelian randomization (MR) study to assess the causal nature of the associations of those ILs with the risk of CAD, AF and ischemic stroke and its subtypes by using summary-level data from published GWASs. As secondary analyses, we investigated the associations of circulating ILs with lipids (low- and high- density lipoprotein cholesterol, total cholesterol and triglycerides), which are potential mediators of any association between ILs and CVD [15].

2. Methods

2.1. Study design

The MR design is based on instrumental variable analysis and can strengthen the inference on the casual nature of associations between risk factors and outcomes by exploiting genetic variants as instrumental variables of an exposure [27]. This technique minimizes confounding as genetic variants are randomly assorted at conception and are generally unrelated to self-selected lifestyle factors, behaviors and environmental factors. In addition, it overcomes reverse causality as allelic randomization antedates the onset of disease. Fig. 1 summarizes the assumptions of the MR design and the data sources used in the current two-sample MR study based on summary-level data from published GWASs on ILs [23–26], cardiovascular disease [28–30], and lipid levels [31]. More details of the GWASs are shown in Supplementary Table 1.

Studies included in the original GWASs had been approved by a relevant institutional review board and participants had provided informed consent. The present analyses have been approved by the Swedish Ethical Review Authority.

2.2. SNP selection

One SNP for IL-2ra, IL-16, and IL-17, two SNPs for IL-1β, IL-1ra and IL-6, and three SNPs for IL-18 were selected at the genome-wide significance level ($P < 5 \times 10^{-8}$) from the summary-level data of three GWASs on these traits [23–26]. The two SNPs associated with IL-1β were in complete linkage disequilibrium ($R^2 = 1.0$). Thus, we included only the SNP with the strongest association with IL-1β. For IL-6, rs643434 in the *ABO* gene was excluded due to strong pleiotropic associations with cardiometabolic traits (34), leaving rs4129267 (in the *IL6R* gene) as instrumental variable for IL-6 in the primary analyses. The SNPs influencing IL-1ra were on the same chromosome but in different gene regions and nearly independent ($R^2 = 0.1$ in the CEU population). The SNPs influencing IL-18 were on different chromosomes and uncorrelated. The phenotypic variance explained by the selected SNPs was about 1.0% for IL-1β, 2.0% for IL-1ra, 4.4% for IL-2ra, 0.5% for IL-6, 4.1% for IL-16, 0.5% for IL-17 and 6.6% for IL-18.

2.3. Outcome sources

Summary-level data for CAD, AF and ischemic stroke and its subtypes were obtained from the Coronary ARtery DIsease Genome-wide Replication and Meta-analysis plus The Coronary Artery Disease Genetics (CARDIoGRAMplusC4D) consortium (60,801 CAD cases and 123,504 non-cases) [28], Atrial Fibrillation consortium (60,801 AF cases and 123,504 non-cases) [29] and MEGASTROKE consortium (34,217 cases of any ischemic stroke, 4373 large artery stroke cases, 7193 cardioembolic stroke cases, 5386 small vessel stroke cases and 404,630 non-cases) [30]. The CAD case status was defined by an inclusive CAD

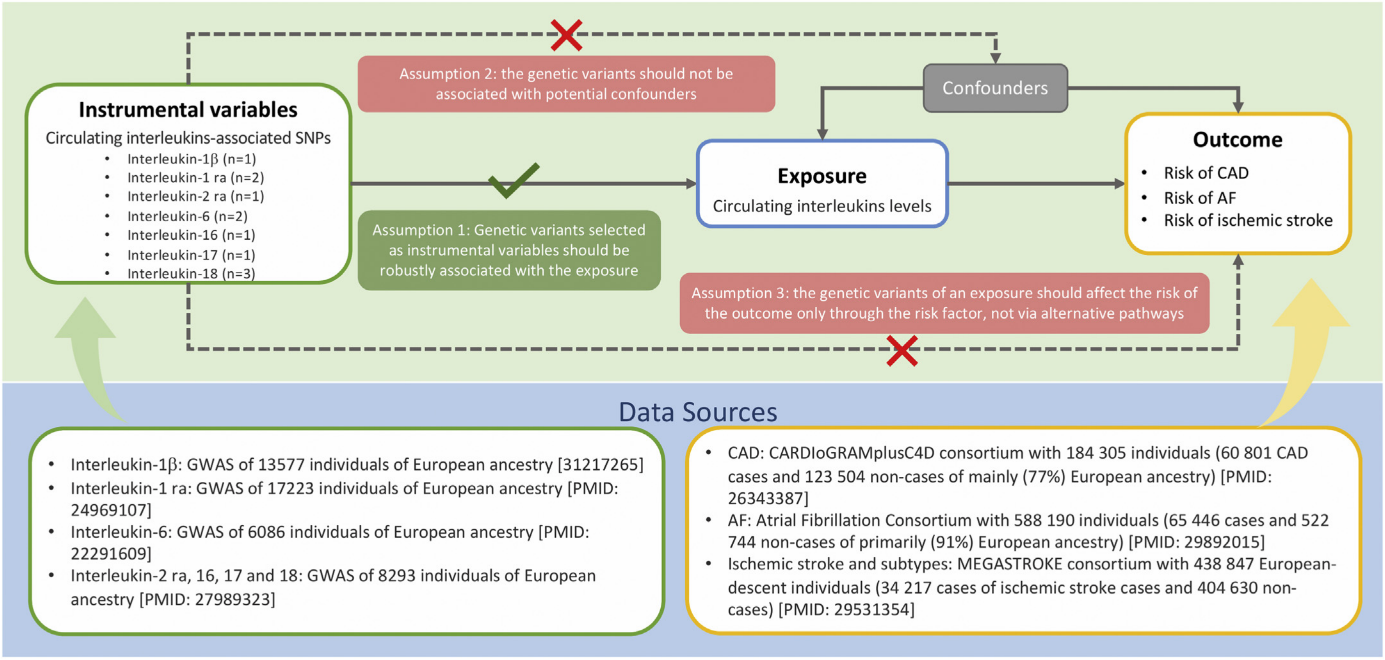

Fig. 1. Assumptions of the Mendelian randomization design and summary of data sources used in the present study. AF indicates atrial fibrillation; CAD, coronary artery disease; GWAS, genome-wide association study.

diagnosis (e.g., myocardial infarction, acute coronary syndrome, chronic stable angina, or coronary stenosis >50%). For AF, cases were defined as individuals with paroxysmal or permanent AF or atrial flutter. The classification of any ischemic stroke and its subtypes was mainly based on the Trial of Org 10,172 in Acute Stroke Treatment (TOAST) criteria. The associations of the selected SNPs for ILs with the CVDs are presented in Supplementary Tables 2 and 3. The IL-1β variant was not available in the CARDIoGRAMplusC4D and MEGASTROKE consortia and no suitable proxy (defined as $R^2 > 0.8$) was identified.

Summary-level data for serum lipid levels were available from Global Lipids Genetics Consortium, including 188,577 European-descent individuals [31]. Four of the IL-related SNPs were not available in the dataset of Global Lipids Genetics Consortium. The proxy SNPs were used by searching the database of Division of Cancer Epidemiology & Genetics in National Cancer Institute at the correlation level of $R^2 \geq 0.8$ [32]. Three of four missing SNPs were replaced by proxies and one SNP for IL-18 were not available and excluded from the analyses. The associations of the selected SNPs for ILs with serum lipids are presented in Supplementary Table 4.

2.4. Statistical analyses and pleiotropy assessment

The IL–outcome associations were estimated using the Wald method [33], and was calculated by dividing the beta coefficient for the SNP–outcome association with the beta coefficient for the SNP–IL association. The inverse-variance weighted method with fixed effects was used to combine the Wald estimates for the two and three SNPs for IL-1ra and IL-18, respectively. The odds ratios (OR) with 95% confidence intervals (CI) of CVD and changes of serum lipids levels were scaled to one standard deviation (SD) increment of genetically predicted circulating levels of ILs. A sensitivity analysis was conducted for IL-6 where the IL-6-related SNP in the *ABO* (blood group) locus was included in the analysis. Power calculation was carried out using a web-tool [34]. All statistical analyses were two-sided and performed in Stata/SE 15.0. To account for multiple testing, we considered associations with P values <0.001 (where $P = 0.05/36$ (six ILs and six outcomes)) to represent strong evidence of causal associations. Associations with P values between 0.05 and 0.001 were considered suggestive evidence of causal associations. We searched the PhenoScanner V2 database (a database of human genotype-phenotype associations) to detect possible pleiotropy for each included SNP [35].

3. Results

3.1. Statistical power

We had over 95% power to detect an OR of 0.7 (or 1.3) for all included circulating ILs and an OR of 0.9 (or 1.1) for IL-2ra, IL-16 and IL-18 in the analyses of CAD, AF and any ischemic stroke (Supplementary Table 5). The power was low in the analyses of ischemic stroke subtypes (Supplementary Table 5).

3.2. ILs and CVD

Genetic predisposition to higher levels of IL-1ra was significantly associated with higher odds of CAD and suggestively associated with higher odds of cardioembolic stroke but was not associated with the other CVDs (Fig. 2). For one SD increase of circulating IL-1ra levels, the ORs were 1.36 (95% confidence interval (CI), 1.14–1.63; $P = 5.37 \times 10^{-4}$) for CAD and 1.44 (95% CI, 1.02–2.03; $P = 0.037$) for cardioembolic stroke.

Genetically predicted circulating IL-6 levels were significantly inversely associated with CAD and AF and suggestively inversely associated with any ischemic stroke, cardioembolic stroke, and small vessel stroke (Fig. 3). The ORs per one SD increase in IL-6 levels were 0.64 (95% CI, 0.54–0.76; $P = 2.22 \times 10^{-7}$) for CAD, 0.70 (95% CI, 0.62–0.80; $P = 1.34 \times 10^{-7}$) for AF, 0.81 (95% CI, 0.67–0.98; $P = 0.026$) for any ischemic stroke, 0.64 (95% CI, 0.45–0.91; $P = 0.013$) for cardioembolic stroke, and 0.65 (95% CI, 0.42–0.99; $P = 0.046$) for small vessel stroke. Results were consistent for all outcomes except small vessel stroke when the IL-6-related SNP in the *ABO* gene was included in the analysis (Supplementary Table 6).

There was a suggestive inverse association between IL-16 levels and CAD (OR per one SD increase in IL-16 levels 0.95; 95% CI, 0.91–1.00; $P = 0.033$) (Supplementary Fig. 1). Genetically predicted IL-1β, IL-2ra, IL-17 and IL-18 levels were not associated with any type of CVD (Supplementary Fig. 1 and Supplementary Fig. 2).

3.3. ILs and serum lipids

Genetic predisposition to higher circulating IL-1ra levels was associated with higher levels of serum low-density lipoprotein cholesterol, total cholesterol and triglycerides, but was not associated with high-density lipoprotein cholesterol (Supplementary Fig. 3). Other ILs were not associated with serum lipid levels (Supplementary Fig. 3).

3.4. Potential pleiotropic associations

The related traits for each included SNP in the present study were searched at the genome-wide significance level and was presented in the Supplementary Table 7. Rs4129267 for IL-6 was associated with blood protein levels, C-reactive protein, fibrinogen (IL-6 as a main regulator of fibrinogen synthesis), allergic disease, rheumatoid arthritis, and asthma. Rs4778636 for IL-16 was associated with blood protein levels. Rs1530455 for IL-17 and rs4251961 for IL-1ra were associated with certain immune cell counts.

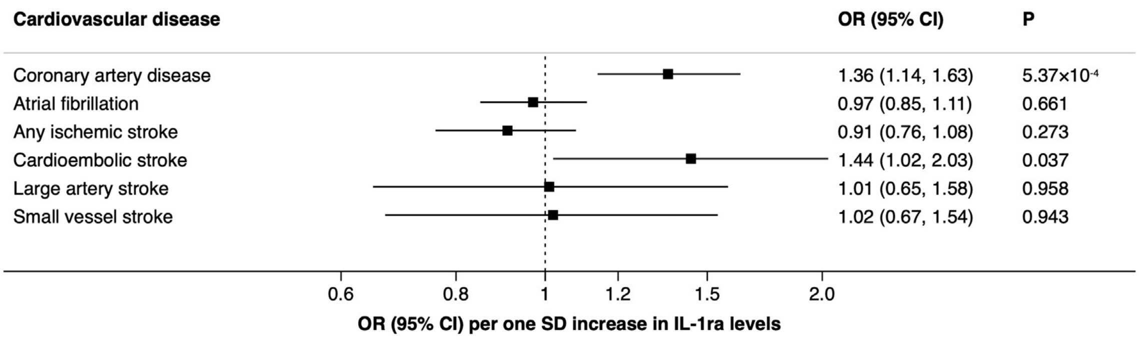

Fig. 2. Associations of genetically predicted IL-1ra levels with cardiovascular disease. CI indicates confidence interval; IL-1ra, interleukin 1 receptor antagonist; OR, odds ratio; SD, standard deviation.

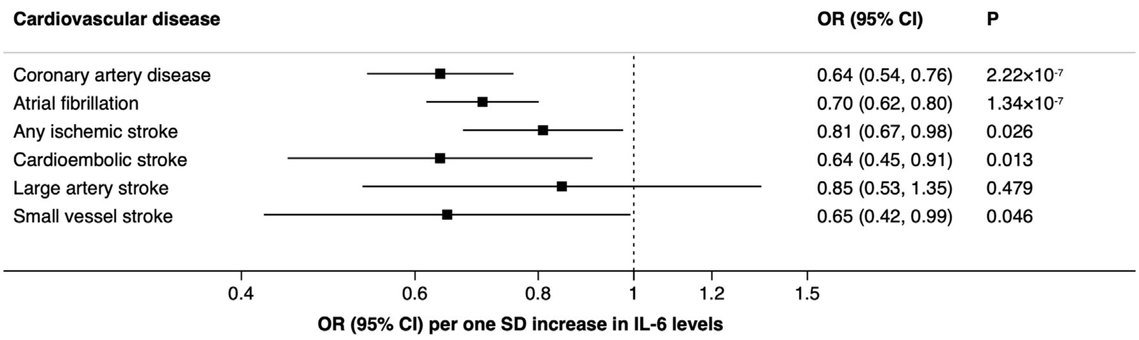

Fig. 3. Associations of circulating IL-6 with cardiovascular disease risk in Mendelian randomization analyses. CI indicates confidence interval; IL-6, interleukin 6; OR, odds ratio; SD, standard deviation.

4. Discussion

The present study showed that genetically predicted IL-1ra levels were positively associated with CAD and cardioembolic stroke, whereas genetically predicted IL-6 levels were inversely associated with CAD, AF, and ischemic stroke, especially cardioembolic and small vessel stroke. There was suggestive evidence of an inverse association between higher IL-16 levels and CAD. The other assessed circulating ILs were not associated with any CVD outcome.

The impact of IL-1ra on CVD risk has been studied during the last century. However, data on cardiovascular effects of IL-1 have been inconsistent. Pre-clinical and clinical studies proved the efficiency of IL-1 blockade on reduction of recurrent atherosclerotic cardiovascular events in patients with prior acute myocardial infarction and elevated C-reactive protein levels [36]. Nevertheless, in a systematic review of five cohort studies with 1855 CVD cases and 18,745 non-cases, circulating IL-1ra levels were positively associated with CVD risk after adjustment of confounders [37]. A clinical study with 499 patients showed that patients with lower IL-1 levels (IL-1(−) genotypes) had lower risk of CAD compared with those with IL-1(+) genotypes, and that the association was mediated by oxidized phospholipids and lipoproteins [38]. In contrast, a large MR study found that dual IL-1α/β inhibition, represented by a genetic score of two SNPs (rs6743376 and rs1542176), increased the risk of CAD by 3% per additional allele [15]. This finding was verified by the present MR study using two different instrumental variables for IL-1ra (rs4251961 and rs6759676) identified at genome-wide significance in a large GWAS [23]. In addition, results from both the previous study [15] and our study showed that genetically higher IL-1ra levels were associated with higher levels of serum lipids, potentially mediating the positive association between IL-1ra and CAD [23].

The evidence on the association between IL-1ra and ischemic stroke is conflicting. A genetic study with 844 ischemic stroke patients and 668 controls found that rs380092 in IL-1ra controlling gene, which is not in linkage equilibrium with the two SNPs used as instrumental variables in the present study, was associated with any ischemic stroke [39]. However, in two small case-control studies, IL-1β polymorphism (−511) was not associated with any ischemic stroke in one of the studies [40] but was associated with small vessel stroke in the other study [41]. In the present MR study, genetic predisposition to higher levels of IL-1ra was associated with suggestive higher odds of cardioembolic stroke but was not associated with any ischemic stroke, large artery stroke, or small vessel stroke. Our null finding for any ischemic stroke is consistent with results of another large MR study of 12,389 ischemic stroke cases and 62,004 controls [15]. Discrepancies between studies may be attributed by different SNP selection and statistical power to detect weak associations between IL-1ra levels and ischemic stroke subtypes. The null association between IL-1ra levels and AF in the present study is in line with the results of an MR study with 6707 AF cases [15].

Our findings for IL-6 are in line with those of previous MR studies of the association between a functional variant in the *IL6R* gene and CAD [12,13]. Those MR studies showed that the variant in the *IL6R* gene did not only associate with higher IL-6 levels and higher odds of CAD but was also associated with lower levels of C-reactive protein and fibrinogen [12,13], effects that are concordant with IL6R blockade by Tocilizumab treatment. As for the effect of IL-6 on AF, most previous observational studies found that high IL-6 levels were associated with the presence and duration of AF and increased left atrial diameter [42]. Previous data on the association between IL-6 genetic variations and ischemic stroke risk were conflicting, possibly because of small sample sizes and different proportions of stroke subtypes in different populations [14]. This MR study is to the best of our knowledge the first to show a potential causal association between IL-6 and risk of ischemic stroke, in particular cardioembolic and small vessel stroke. The association of genetically higher IL-6 levels with lower odds of CAD, AF, and ischemic stroke may seem paradoxical. However, a possible explanation is that higher levels of circulating IL-6 correspond to lower cellular binding of IL-6 to its receptor and lower bio-function of IL-6, implying impaired IL-6 signaling [12]. Therefore, our findings indicate that IL-6 inhibition is associated with lower risk of CVD.

Few studies have examined the associations of IL-16, IL-17 and IL-18 with CAD, AF and ischemic stroke. In one study with 157 CAD patients and 202 healthy controls, T allele in one IL-16 controlling gene was associated with a significantly lower odds of CAD compared with G allele in rs11556218 [18], which is consistent with our finding (T allele in rs11556218 is highly correlated with G allele in rs4778636, $R^2 = 0.94$). Another study also found a specific IL-16-related SNP (rs8034928) to be significantly associated with the risk of CAD in a Chinese Han population [19]. High levels of IL-17 [20] and IL-18 [21] were reported to be associated with an increased risk of CAD in two studies, but no causal association between those ILs and CAD was supported by the present MR study. The levels of IL-18 were significantly higher in AF patients undergoing coronary artery bypass surgery [22] or in the absence of structural heart disease [17] compared to non-AF individuals. The present study suggested a borderline positive association between genetically predicted IL-18 levels and AF.

There are several potential explanations for the findings in the present study. With regards to IL-6, the functional polymorphism tagged by rs7529229 (highly correlated with rs4129267; $R^2 = 0.98$) causes increased soluble IL-6R levels by increasing proteolytic cleavage of the membrane-bound receptor [12]. The elevated soluble IL-6R levels acts on a diverse range of cell types including megakaryocytes and endothelial cells, thereby influencing the risk of CAD [12]. In addition, levels of IL-6 influence the risk of CVD possibly through its negative feedback on IL-1 and tumor necrosis factor alpha that initiate the inflammatory cascade, especially for patients after stroke [14]. IL-6 also links with the regulation of C-reactive protein, fibrinogen, and serum amyloid-A [42]. C-reactive protein does not appear to be causally associated with

CVD risk [43], but the role of other pathways controlled by IL-6 for CVD development needs further research.

The negative effects of IL-1ra on CVD could be explained, at least partly, by their counteractive interaction with the IL-1 signaling pathway that regulates the metabolism of low-density lipoprotein cholesterol and triglycerides, as shown in the present study and other researches [15]. Elevated low-density lipoprotein cholesterol and triglyceride levels are associated with CAD but not cardioembolic stroke [44,45]. The IL-1 signaling pathway was also reported to influence the formation of atheromatous lesions, vascular inflammation and plaque rupture, which are associated with CVD risk [46]. Moreover, activation of inflammasome complex, which is critical for the generation of active IL-1, has been proposed to participate in the development of CVD [47]. With regards to the role of IL-16 in CVD, while it was not well understood about the underlying mechanisms, some studies prompted a regulatory effect of IL-16 on CAD development by promoting cardiac fibrosis and myocardial stiffening [48]. Apart from that, the infiltration of T cells to human epicardial adipose tissue was demonstrated to be inversely associated with CAD [49], which suggested a role of IL-16 during this biological process given the fact that IL-16 is a major chemoattractant of T cells.

A major strength of this study is the MR approach, which minimized confounding and reverse causality. In addition, we systematically tested the association of ILs levels with several cardiovascular diseases using summary-level data from large-scale genetic consortia. Hence, we had high statistical power to detect weak associations of ILs with CAD, AF and total ischemic stroke but not ischemic stroke subtypes due to limited number of cases. We restricted the study population to European ancestry as much as possible to reduce bias from population stratification. However, this restriction reduced the transferability to individuals of non-European descents. In addition, the genetic instrument for IL-6 was extracted from a GWAS with mere Sardinians ancestry. However, another SNP (rs7529229) in linkage disequilibrium ($R^2 = 0.98$) with the selected SNP for IL-6 in the *IL6R* gene region has been shown to be strongly associated with IL-6 levels ($P = 8.41 \times 10^{-68}$) in 29,838 individuals of European ancestry from 17 studies [12]. Another limitation of this study is that we only had three or less SNPs as instrumental variables for individual ILs. This confined sensitivity analyses to explore potential pleiotropic effects, which might weaken the reliability of the results. However, we detected no possible pleiotropic effects, driving the associations, for the SNP included in the present study.

5. Conclusions

This MR study strengthens the evidence that IL-6 inhibition may offer a therapeutic approach for prevention of CAD, AF, and ischemic stroke. In contrast, IL-1 inhibition through raised IL-1ra levels may confer increased risk of CAD and cardioembolic stroke. The role of IL-16 for CAD warrants further investigation.

Acknowledgments

Summary-level data for genetic associations with the CAD, AF, ischemic stroke and lipids have been contributed by the CARDIoGRAMplusC4D consortium, Atrial Fibrillation consortium, MEGASTROKE consortium and Global Lipids Genetics Consortium. The authors thank all investigators for sharing these data. SY and SCL designed the study and analyzed the data. SY drafted the manuscript. SY, AL, QQH, SB, and SCL interpreted the data and reviewed the paper. All authors provided the corresponding author with permission to be named in the manuscript.

Sources of funding

This work was supported by the Swedish Research Council for Health, Working Life, and Welfare, (Grant Number 2018-00123) the Swedish Research Council (Grant Number 2019-00977), and the Swedish Heart-Lung Foundation (Grant Number 20190247). SB is supported by a Sir Henry Dale Fellowship jointly funded by the Wellcome Trust and the Royal Society (Grant Number 204623/Z/16/Z). None of the authors has any conflicts of interest.

Declaration of competing interest

None.

Appendix A. Supplementary data

Supplementary data to this article can be found online at https://doi.org/10.1016/j.ijcard.2020.03.053.

References

[1] Susanna C. Larsson MB, Jessica M.B. Rees, Amy M. Mason, Stephen Burgess, Body mass index and body composition in relation to 14 cardiovascular conditions in UK Biobank: a Mendelian randomization study, Eur. Heart J. 41 (2019) 221–226.
[2] M.V. Holmes, C.E. Dale, L. Zuccolo, R.J. Silverwood, Y. Guo, Z. Ye, et al., Association between alcohol and cardiovascular disease: Mendelian randomisation analysis based on individual participant data, BMJ 349 (2014), g4164.
[3] A. Wahid, N. Manek, M. Nichols, P. Kelly, C. Foster, P. Webster, et al., Quantifying the association between physical activity and cardiovascular disease and diabetes: a systematic review and meta-analysis, J. Am. Heart Assoc. 5 (2016)(pii: e002495).
[4] S.C. Larsson, S. Burgess, K. Michaelsson, Serum magnesium levels and risk of coronary artery disease: Mendelian randomisation study, BMC Med. 16 (2018) 68.
[5] L. Wang, J.E. Manson, Y. Song, H.D. Sesso, Systematic review: vitamin D and calcium supplementation in prevention of cardiovascular events, Ann. Intern. Med. 152 (2010) 315–323.
[6] P. Maffia, G. Cirino, Targeting inflammation to reduce cardiovascular disease risk, Br. J. Pharmacol. 174 (2017) 3895–3897.
[7] J.F. Rossi, Z.Y. Lu, M. Jourdan, B. Klein, Interleukin-6 as a therapeutic target, Clin. Cancer Res. 21 (2015) 1248–1257.
[8] S. Onuora, Rheumatoid arthritis: Sarilumab more effective than adalimumab, Nat. Rev. Rheumatol. 13 (2017) 2.
[9] C. Salliot, M. Dougados, L. Gossec, Risk of serious infections during rituximab, abatacept and anakinra treatments for rheumatoid arthritis: meta-analyses of randomised placebo-controlled trials, Ann. Rheum. Dis. 68 (2009) 25–32.
[10] J. Danesh, S. Kaptoge, A.G. Mann, N. Sarwar, A. Wood, S.B. Angleman, et al., Long-term interleukin-6 levels and subsequent risk of coronary heart disease: two new prospective studies and a systematic review, PLoS Med. 5 (2008), e78.
[11] S. Kaptoge, S.R. Seshasai, P. Gao, D.F. Freitag, A.S. Butterworth, A. Borglykke, et al., Inflammatory cytokines and risk of coronary heart disease: new prospective study and updated meta-analysis, Eur. Heart J. 35 (2014) 578–589.
[12] D.I. Swerdlow, M.V. Holmes, K.B. Kuchenbaecker, J.E. Engmann, T. Shah, R. Sofat, et al., The interleukin-6 receptor as a target for prevention of coronary heart disease: a mendelian randomisation analysis, Lancet. 379 (2012) 1214–1224.
[13] N. Sarwar, A.S. Butterworth, D.F. Freitag, J. Gregson, P. Willeit, D.N. Gorman, et al., Interleukin-6 receptor pathways in coronary heart disease: a collaborative meta-analysis of 82 studies, Lancet. 379 (2012) 1205–1213.
[14] A.R. Tso, J.G. Merino, S. Warach, Interleukin-6 174G/C polymorphism and ischemic stroke: a systematic review, Stroke. 38 (2007) 3070–3075.
[15] Cardiometabolic effects of genetic upregulation of the interleukin 1 receptor antagonist: a Mendelian randomisation analysis, Lancet Diabetes Endocrinol. 3 (2015) 243–253.
[16] C.J. Smith, S. Hulme, A. Vail, C. Heal, A.R. Parry-Jones, S. Scarth, et al., SCIL-STROKE (subcutaneous Interleukin-1 receptor antagonist in ischemic stroke): a randomized controlled phase 2 trial, Stroke. 49 (2018) 1210–1216.
[17] Y. Luan, Y. Guo, S. Li, B. Yu, S. Zhu, N. Li, et al., Interleukin-18 among atrial fibrillation patients in the absence of structural heart disease, Europace. 12 (2010) 1713–1718.
[18] J. Wu, Y. Wang, Y. Zhang, L. Li, Association between interleukin-16 polymorphisms and risk of coronary artery disease, DNA Cell Biol. 30 (2011) 305–308.
[19] H. Huang, Z. Zeng, L. Zhang, R. Liu, X. Li, O. Qiang, et al., The association of interleukin-16 gene polymorphisms with susceptibility of coronary artery disease, Clin. Biochem. 46 (2013) 241–244.
[20] S. Hashmi, Q.T. Zeng, Role of interleukin-17 and interleukin-17-induced cytokines interleukin-6 and interleukin-8 in unstable coronary artery disease, Coron. Artery Dis. 17 (2006) 699–706.
[21] S. Blankenberg, G. Luc, P. Ducimetiere, D. Arveiler, J. Ferrieres, P. Amouyel, et al., Interleukin-18 and the risk of coronary heart disease in European men: the prospective epidemiological study of myocardial infarction (PRIME), Circulation. 108 (2003) 2453–2459.
[22] Z.K. Wu, J. Laurikka, S. Vikman, R. Nieminen, E. Moilanen, M.R. Tarkka, High postoperative interleukin-8 levels related to atrial fibrillation in patients undergoing coronary artery bypass surgery, World J. Surg. 32 (2008) 2643–2649.
[23] C. Herder, M.L. Nuotio, S. Shah, S. Blankenberg, E.J. Brunner, M. Carstensen, et al., Genetic determinants of circulating interleukin-1 receptor antagonist levels and their association with glycemic traits, Diabetes. 63 (2014) 4343–4359.

[24] S. Naitza, E. Porcu, M. Steri, D.D. Taub, A. Mulas, X. Xiao, et al., A genome-wide association scan on the levels of markers of inflammation in Sardinians reveals associations that underpin its complex regulation, PLoS Genet. 8 (2012), e1002480.
[25] A.V. Ahola-Olli, P. Wurtz, A.S. Havulinna, K. Aalto, N. Pitkanen, T. Lehtimaki, et al., Genome-wide association study identifies 27 loci influencing concentrations of circulating cytokines and growth factors, Am. J. Hum. Genet. 100 (2017) 40–50.
[26] E. Sliz, M. Kalaoja, A. Ahola-Olli, O. Raitakari, M. Perola, V. Salomaa, et al., Genome-wide association study identifies seven novel loci associating with circulating cytokines and cell adhesion molecules in Finns, J. Med. Genet. 56 (2019) 607–616.
[27] G.D. Smith, S. Ebrahim, 'Mendelian randomization': can genetic epidemiology contribute to understanding environmental determinants of disease? Int. J. Epidemiol. 32 (2003) 1–22.
[28] M. Nikpay, A. Goel, H.H. Won, L.M. Hall, C. Willenborg, S. Kanoni, et al., A comprehensive 1,000 genomes-based genome-wide association meta-analysis of coronary artery disease, Nat. Genet. 47 (2015) 1121–1130.
[29] C. Roselli, M.D. Chaffin, L.C. Weng, S. Aeschbacher, G. Ahlberg, C.M. Albert, et al., Multi-ethnic genome-wide association study for atrial fibrillation, Nat. Genet. 50 (2018) 1225–1233.
[30] R. Malik, G. Chauhan, M. Traylor, M. Sargurupremraj, Y. Okada, A. Mishra, et al., Multiancestry genome-wide association study of 520,000 subjects identifies 32 loci associated with stroke and stroke subtypes, Nat. Genet. 50 (2018) 524–537.
[31] C.J. Willer, E.M. Schmidt, S. Sengupta, G.M. Peloso, S. Gustafsson, S. Kanoni, et al., Discovery and refinement of loci associated with lipid levels, Nat. Genet. 45 (2013) 1274–1283.
[32] M.J. Machiela, S.J. Chanock, LDlink: a web-based application for exploring population-specific haplotype structure and linking correlated alleles of possible functional variants, Bioinformatics. 31 (2015) 3555–3557.
[33] S. Burgess, D.S. Small, S.G. Thompson, A review of instrumental variable estimators for Mendelian randomization, Stat. Methods Med. Res. 26 (2017) 2333–2355.
[34] M.J. Brion, K. Shakhbazov, P.M. Visscher, Calculating statistical power in Mendelian randomization studies, Int. J. Epidemiol. 42 (2013) 1497–1501.
[35] M.A. Kamat, J.A. Blackshaw, R. Young, P. Surendran, S. Burgess, J. Danesh, et al., PhenoScanner V2: an expanded tool for searching human genotype-phenotype associations, Bioinformatics. 35 (2019) 4851–4853.
[36] L.F. Buckley, A. Abbate, Interleukin-1 blockade in cardiovascular diseases: a clinical update, Eur. Heart J. 39 (2018) 2063–2069.
[37] C. Herder, T. de Las Heras Gala, M. Carstensen-Kirberg, C. Huth, A. Zierer, S. Wahl, et al., Circulating levels of interleukin 1-receptor antagonist and risk of cardiovascular disease: meta-analysis of six population-based cohorts, Arterioscler. Thromb. Vasc. Biol. 37 (2017) 1222–1227.
[38] S. Tsimikas, G.W. Duff, P.B. Berger, J. Rogus, K. Huttner, P. Clopton, et al., Pro-inflammatory interleukin-1 genotypes potentiate the risk of coronary artery disease and cardiovascular events mediated by oxidized phospholipids and lipoprotein(a), J. Am. Coll. Cardiol. 63 (2014) 1724–1734.
[39] S. Olsson, L. Holmegaard, K. Jood, M. Sjogren, G. Engstrom, H. Lovkvist, et al., Genetic variation within the interleukin-1 gene cluster and ischemic stroke, Stroke. 43 (2012) 2278–2282.
[40] T. Dziedzic, A. Slowik, J. Pera, A. Szczudlik, Lack of association between interleukin-1 beta polymorphism (−511) and ischaemic stroke, J. Neurol. Neurosurg. Psychiatry 75 (2004) 170–171.
[41] T. Dziedzic, A. Slowik, J. Pera, A. Szczudlik, Interleukin 1 beta polymorphism (−511) and risk of stroke due to small vessel disease, Cerebrovasc. Dis. 20 (2005) 299–303.
[42] Y. Guo, G.Y. Lip, S. Apostolakis, Inflammation in atrial fibrillation, J. Am. Coll. Cardiol. 60 (2012) 2263–2270.
[43] B.P. Prins, A. Abbasi, A. Wong, A. Vaez, I. Nolte, N. Franceschini, et al., Investigating the causal relationship of C-reactive protein with 32 complex somatic and psychiatric outcomes: a large-scale cross-consortium Mendelian randomization study, PLoS Med. 13 (2016), e1001976.
[44] G. Hindy, G. Engstrom, S.C. Larsson, M. Traylor, H.S. Markus, O. Melander, et al., Role of blood lipids in the development of ischemic stroke and its subtypes: a Mendelian randomization study, Stroke. 49 (2018) 820–827.
[45] M.V. Holmes, F.W. Asselbergs, T.M. Palmer, F. Drenos, M.B. Lanktree, C.P. Nelson, et al., Mendelian randomization of blood lipids for coronary heart disease, Eur. Heart J. 36 (2015) 539–550.
[46] B.W. Van Tassell, S. Toldo, E. Mezzaroma, A. Abbate, Targeting interleukin-1 in heart disease, Circulation. 128 (2013) 1910–1923.
[47] L.E. Pavillard, F. Marin-Aguilar, P. Bullon, M.D. Cordero, Cardiovascular diseases, NLRP3 inflammasome, and western dietary patterns, Pharmacol. Res. 131 (2018) 44–50.
[48] J.A. Diaz, A.J. Booth, G. Lu, S.C. Wood, D.J. Pinsky, D.K. Bishop, Critical role for IL-6 in hypertrophy and fibrosis in chronic cardiac allograft rejection, Am. J. Transplant. 9 (2009) 1773–1783.
[49] M. Mraz, A. Cinkajzlova, J. Klouckova, Z. Lacinova, H. Kratochvilova, M. Lips, et al., Coronary artery disease is associated with an increased amount of T lymphocytes in human epicardial adipose tissue, Mediat. Inflamm. 2019 (2019) 4075086.
